# Psychosocial stress reactivity habituates following acute physiological stress

**DOI:** 10.1002/hbm.25106

**Published:** 2020-06-29

**Authors:** Anne Kühnel, Nils B. Kroemer, Immanuel G. Elbau, Michael Czisch, Philipp G. Sämann, Martin Walter, BeCome working group, Elisabeth B. Binder

**Affiliations:** ^1^ Department of Translational Research in Psychiatry Max Planck Institute of Psychiatry Munich Germany; ^2^ International Max Planck Research School for Translational Psychiatry (IMPRS‐TP) Munich Germany; ^3^ Department of Psychiatry and Psychotherapy University of Tübingen Tübingen Germany; ^4^ Max Planck Institute of Psychiatry Munich Germany; ^5^ Leibniz Institute for Neurobiology Magdeburg Germany; ^6^ Department of Psychiatry and Psychotherapy Otto‐von‐Guericke University Magdeburg Magdeburg Germany; ^7^ Department of Psychiatry and Psychotherapy Jena University Hospital Jena Germany

**Keywords:** cortisol, fMRI, habituation, HPA axis, representational similarity, stress

## Abstract

Acute and chronic stress are important factors in the development of mental disorders. Reliable measurement of stress reactivity is therefore pivotal. Critically, experimental induction of stress often involves multiple “hits” and it is an open question whether individual differences in responses to an earlier stressor lead to habituation, sensitization, or simple additive effects on following events. Here, we investigated the effect of the individual cortisol response to intravenous catheter placement (IVP) on subsequent neural, psychological, endocrine, and autonomous stress reactivity. We used an established psychosocial stress paradigm to measure the acute stress response (*Stress*) and recovery (*PostStress*) in 65 participants. Higher IVP‐induced cortisol responses were associated with lower pulse rate increases during stress recovery (*b* = −4.8 bpm, *p* = .0008) and lower increases in negative affect after the task (*b* = −4.2, *p* = .040). While the cortisol response to IVP was not associated with subsequent specific stress‐induced neural activation patterns, the similarity of brain responses *Pre‐* and *PostStress* was higher IVP‐cortisol responders (*t*[64] = 2.35, *p* = .022) indicating faster recovery. In conclusion, preparatory stress induced by IVP reduced reactivity in a subsequent stress task by modulating the latency of stress recovery. Thus, an individually stronger preceding release of cortisol may attenuate a second physiological response and perceived stress suggesting that relative changes, not absolute levels are crucial for stress attribution. Our study highlights that considering the entire trajectory of stress induction during an experiment is important to develop reliable individual biomarkers.

## Graphical abstract

Stress often involves multiple “hits” and it is an open question whether individual differences in responses to an earlier stressor lead to habituation, sensitization, or additive effects. Here, we show that a cortisol response to the placement of an intravenous catheter attenuated reactivity to a subsequent psychosocial stress task on the neural, endocrine, autonomous, and subjective level.

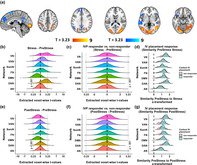

## 1 INTRODUCTION

Acute stress and possible maladaptive responses such as increased anxiety, extensive rumination and impaired cognitive functioning (Mizoguchi et al., 2000) are important factors in the etiology of affective disorders (McEwen, 2004). An important biomarker quantifying the stress response and linking it to personality traits and disease is the hypothalamus–pituitary–adrenal (HPA) axis response to standardized stress tests (Foley & Kirschbaum, 2010), such as the Trier Social Stress Task (TSST) (Kirschbaum, Pirke, & Hellhammer, 1993) and adaptations suitable for exploration through functional imaging (fMRI) (Elbau et al., 2018; Noack, Nolte, Nieratschker, Habel, & Derntl, 2019). Whereas stress is an integral part of everyday life, responding to repeated stressful experiences can unveil inter‐individual differences that have been linked to psychopathology before (Grillon, Southwick, & Charney, 1996; McEwen, 1998; McLaughlin, Conron, Koenen, & Gilman, 2010). Decades of preclinical and human research have demonstrated the interdependence of multiple stressful events often leading to habituation or sensitization of acute responses to impending stress (Belda, Fuentes, Daviu, Nadal, & Armario, 2015; Grissom & Bhatnagar, 2009; Petrowski, Wintermann, & Siepmann, 2012; Pitman, Ottenweller, & Natelson, 1990). However, there is little evidence from multimodal experimental studies on potential carry‐over effects of directly preceding stress suggesting an implicit assumption that sequential stress effects are independent and additive. Here, we sought to bridge this gap by investigating inter‐individual differences in cortisol responses to a stressful precedence (here: placement of an intravenous catheter) on the experience of psychosocial stress.

Consequently, the basal state of the stress system at the time of the stressor plays an important role in modulating endocrine stress responses (Dickerson & Kemeny, 2004; Juster, Perna, Marin, Sindi, & Lupien, 2012; Kudielka, Schommer, Hellhammer, & Kirschbaum, 2004). Alterations in the basal HPA axis state may even affect the cognitive appraisal of the stress‐induced physiological changes, thereby altering the emotional response (Folkman, Lazarus, Dunkel‐Schetter, DeLongis, & Gruen, 1986; Ursin & Eriksen, 2004). The TSST has been validated extensively and a number of influencing factors have been characterized to date (Allen, Kennedy, Cryan, Dinan, & Clarke, 2014). For instance, time of day (Kudielka et al., 2004), timing of cortisol measurements (Dickerson & Kemeny, 2004; Liu et al., 2017), composition and feedback (e.g., neutral vs. negative) of the panel, sex and menstrual cycle (Childs, Dlugos, & Wit, 2010; Liu et al., 2017) have been shown to impact stress reactivity. One crucial factor that may influence basal states is the intravenous catheter placement (IVP) for the repeated assessment of serum cortisol levels (Dickerson & Kemeny, 2004; Goodman, Janson, & Wolf, 2017; Kudielka et al., 2004). Experimental evidence for the importance of the basal HPA axis state comes from studies showing that a pharmacological increase of cortisol before the TSST reduced subjective stress after the task (Het & Wolf, 2007). Similarly, endogenous cortisol increases induced by either physical exercise or anticipation of a stress task or the MRI were associated with a reduced endocrine or physiological response to the psychosocial stressor, albeit at a group level (Gossett et al., 2018; Juster et al., 2012; Zschucke, Renneberg, Dimeo, Wüstenberg, & Ströhle, 2015). Likewise, reduced cortisol responses to the TSST as a result of two subsequent sessions on the same day indicate that biological habituation of the HPA axis may be relevant for repeated stressors in a short time window (Höhne et al., 2014). Lasting effects of cortisol have also been described for functional connectivity at rest (Vaisvaser et al., 2013) and task‐related activity. For example, an unrelated, previously induced cortisol response altered the neural response to the imaging stress task (Zschucke et al., 2015) and other tasks (Maier, Makwana, & Hare, 2015) even up to 60 min later (Joëls, Fernandez, & Roozendaal, 2011). Collectively, this suggests that a preceding acute cortisol response may have lasting effects on the endocrine, physiological, neural and psychological response to subsequent experimental stressors.

To evaluate the interdependence of stressful events and how it may affect potential biomarkers of stress, we quantified the effect of inter‐individual differences in cortisol responses elicited by IVP on the subsequent stress response to a multimodal psychosocial stress test. A recent meta‐analysis (Goodman et al., 2017) showed that cortisol responses to the TSST are indeed influenced by IVP, with effects sizes of the cortisol response being significantly higher in studies with IVP versus without. However, other confounding factors such as interindividual differences in cortisol response to IVP, timing of IVP, or the different methods used to quantify the cortisol response (saliva vs. serum) were not controlled for and effects on other levels of the stress response were not evaluated. Therefore, we first characterized the cortisol response elicited by IVP before a stress task. We then tested if this IVP‐induced increase in cortisol altered the stress response to a subsequent standardized fMRI stress task. Critically, we assessed the stress reactivity on multiple levels including neural (fMRI), autonomous, endocrine, and subjective read‐outs. Moreover, the task was separated into three phases of arithmetic, starting with a control condition without psychosocial stress, followed by the actual psychosocial stressor and ending again with control condition without psychosocial stress. This enabled us to also assess the fast stress recovery during the post stress phase, which may show greater sensitivity to individual stress‐response profiles. We investigated if a stronger preceding IVP‐induced cortisol response would alter the stress response to the subsequent stress task. According to habituation (Gossett et al., 2018; Juster et al., 2012) or sensitization (Goodman et al., 2017) of the stress system, a stronger IVP‐induced cortisol response could either exacerbate or limit the magnitude of a second, task‐induced response.

## 2 METHODS

### 2.1 Participants

The sample was recruited as part of the Biological Classification of Mental Disorders (BeCOME) study at the Max Planck Institute of Psychiatry, registered on ClinicalTrials.gov: NCT03984084. The BeCOME study characterizes participants with a broad spectrum of affective, anxiety, and stress‐related mental disorders as well as unaffected individuals. It includes various behavioral and functional imaging tasks measured across 2 days (Brückl et al., 2020). For the present study we included a subsample of 67 participants (26 women, *M*_age_ = 32.4 years ± 9.7) that contacted the institute as healthy control participants. All participants underwent a comprehensive, computer‐based, standardized diagnostic interview (CIDI) in which diagnoses are derived by an automatically evaluated, standardized, DSM‐IV‐based algorithm. We did not exclude participants that received a diagnosis and thus capture a sample of participants self‐identifying as healthy yet showing symptoms that would be considered subclinical or lead to a diagnosis in multiple cases. Briefly, 48% (*n* = 32) did not have any current or lifetime diagnosis, while 40% (*n* = 27) received at least one (lifetime) diagnosis belonging to the anxiety disorders spectrum including specific phobias, 19% (*n* = 13) a substance use‐related diagnosis and 7% (*n* = 5) a mood disorder (See Table S1 for details). However, none of the participants reported any present medication for their psychiatric symptoms.

To maximize the sample size, we excluded participants with missing or low‐quality data for each analysis separately. More specifically, we excluded participants because of missing cortisol saliva (*n* = 2) samples from all analyses, and serum samples (*n* = 12) from analyses regarding serum cortisol responses to the stress task (both insufficient biological material). Moreover, we excluded 12 participants from analyses regarding pulse rate as their signal quality was too low for reliable peak detection.

### 2.2 Experimental procedure

**FIGURE 1:**
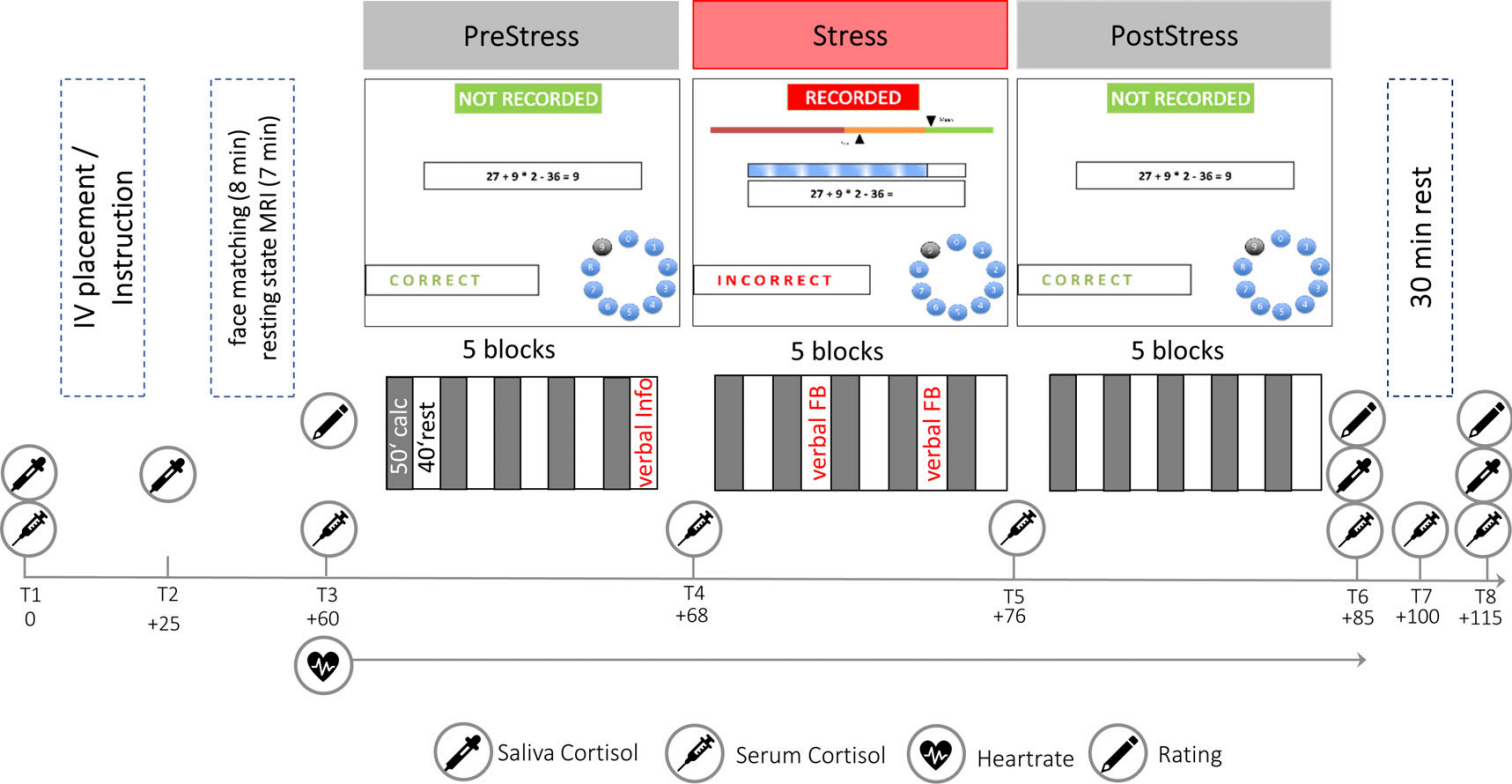
Schematic summary of the procedure and task. Before the stress phase, participants were informed about being recorded in the following trials. Additional aversive verbal feedback (verbal FB) about unsatisfactory performance was given in the second and fourth rest period of the Stress condition. The first serum and saliva samples were taken directly after IV placement

The imaging stress task (Figure 1) was included in the fMRI session on the second BeCOME study day (Brückl et al., 2020). All participants previously took part in the fMRI session on the first study day and consequently none of the participants was fMRI naïve. On this day, participants arrived at the scanner at approximately 10 a.m. Upon arrival, the first saliva sample was taken to measure basal cortisol levels. Subsequently, the IV catheter was placed and tested for permeability for repeated serum sampling measurements during and after the stress task. Problems during the procedure (e.g., failed first or multiple IVP attempts) were recorded by the physicians. After that, participants were familiarized with the task and the response options. Electrodes were placed on the palm of the left hand for the measurement of skin conductance and on the back for electrocardiography. A pulse oximeter was placed on the fingertip to measure pulse rate. Before entering the scanner (21.8 min ± 7.6 after IVP), we took another saliva sample to assess cortisol increases related to the IVP. The fMRI session started with a T2‐weighted high‐resolution image for spatial normalization, followed by an emotional face matching task and a pre‐stress resting state. Immediately before the stress paradigm, participants rated their current affective state using the previously used (Elbau et al., 2018) *Befindensskalierung nach Kategorien und Eigenschaftsworten* (BSKE, for details see Supporting Information). Approximately 60 min (64.6 min ± 8.7) after IVP, the stress task started. A 60‐min interval is generally recommended for recovery of the cortisol concentrations back to baseline after IVP (Allen et al., 2014). The task consisted of a PreStress, Stress, and PostStress phase and lasted for about 25 min. Multiple blood samples were taken during task performance (Figure 1). After completion of the task, the current affective state was assessed again with the BSKE and saliva and blood samples were taken. A 30‐min rest period lying outside the scanner was followed by a concluding assessment of subjective affect, blood and saliva cortisol samples, and post‐task resting state fMRI. At the end of the session, participants were debriefed by the investigator.

### 2.3 Paradigm

Psychosocial stress was induced by an imaging stress task previously reported by Elbau et al. (2018), with minor changes regarding the aversiveness of the feedback and the number of task blocks. As in previous versions of the task, participants had to solve mental arithmetic problems either in a control condition without time pressure and negative feedback or under stress with a time limit and negative feedback. Critically, the task was partitioned into three phases, PreStress, Stress, and PostStress, each consisting of five 50 s blocks of arithmetic interleaved with five 40 s blocks rest (fixation cross). During an arithmetic block, participants were presented an arithmetic problem with a solution between 0 and 9. Arithmetic problems varied in their difficulty across three levels and difficulty was balanced across the three conditions. The correct answer was chosen using a response box allowing to navigate a two‐button dial wheel system. After selecting the answer, the screen “froze” for an anticipation phase (2.5 ± 1 s, jittered) that was followed by the feedback (“correct,” “incorrect” or “timeout”, presented for 660 ms). During PreStress and PostStress, participants had 10.5 s to respond and no further evaluative feedback or cues were given. Before Stress, participants were informed that answers are now “recorded”. During stress, time to solve the arithmetic problem was generally limited to 4.5 s, and in part self‐adaptive depending on the participant's preceding performance. Further, a time bar indicated how much time was left, inducing further time pressure, and a performance indicator showed that current performance was below group average (“in the red area”). Two instances of scripted negative verbal feedback in two rest periods informed the participants about their sub‐par performance and pushed them to work harder.

### 2.4 Data acquisition

#### 2.4.1 Cortisol sampling (serum and saliva) and analysis

Cortisol concentrations were measured repeatedly before, during, and after the task in saliva and/or serum (Figure 1). Salivary cortisol was sampled directly at arrival before IVP (T1), 20 min after IVP to quantify potential effects of the placement itself (T2) and additionally directly after the stress paradigm (T6) and 30 min after the end (T8) using salivettes cortisol code blue with a synthetic swab (Sarstedt AG & Co., Nümbrecht, Germany). After collection, all probes were centrifuged and stored at −80°C until further processing. Salivary cortisol concentrations were measured with electro‐chemiluminescence‐assay (ECLIA) kit (Cobas®, Roche Diagnostics GmbH, Mannheim, Germany). The detection limit was 1,090 pg/ml. The %CV (coefficient of variation) in saliva samples with varying concentrations was between 2.5 and 6.1% for intra‐assay variability and between 3.6 und 11.8% for inter‐assay variability.

To assess the HPA‐axis response to the psychosocial stress task with a higher temporal resolution, we additionally repeatedly measured serum cortisol. It was sampled at seven time points, first directly after IVP and then in 8‐min intervals starting directly before the task and ending after the 30‐min rest period. After collection, all probes were centrifuged and stored at −80°C until further processing. Serum cortisol was determined using an Enzyme‐linked Immunosorbent Assay (ELISA) kit (IBL Hamburg, Germany). The standard range was 20–800 ng/ml. The %CV in serum samples was between 2.6 and 3.5% for intra‐assay variability and between 2.1 und 5% for inter‐assay variability.

#### 2.4.2 Physiological recording and preprocessing

The autonomous stress response was measured throughout the complete task using photoplethysmography (PPG), electrocardiography, and skin conductance. The PPG data was acquired with an MR compatible pulse oximeter (Nonin Medical Inc., Plymouth, MN) attached to the pulp of the left ring finger. PPG data, sampled at 5 kHz, was amplified using a MR compatible multi‐channel BrainVision ExG AUX Box coupled with a BrainVision ExG MR Amplifier (Brain Products GmbH, Gilching, Germany) and recorded with BrainVision Recorder software 1.0. After down‐sampling to 100 Hz, RR‐intervals were detected using the Physionet Cardiovascular Signal toolbox (Vest et al., 2018). Success of detection of beat positions was evaluated by visual inspection. Measurements with insufficient data‐quality leading to failed detection of beat positions were excluded (*n* = 12). Subsequent analysis of the pulse rate was based on the derived RR‐intervals and conducted with the RHRV package (Rodríguez‐Liñares, Vila, Mendez, Lado, & Olivieri, 2008) for R. Further preprocessing involved the exclusion of implausible interbeat‐intervals (IBI). We filtered out IBIs shorter than 0.3 s and longer than 2.4 s and excluded IBIs showing excessive deviations from the previous, following, or running average (50 beats) IBI. The threshold for excessive deviations was updated dynamically with the initial threshold set at 13% change from IBI to IBI (Vila et al., 1997).

#### 2.4.3 fMRI data acquisition and preprocessing

MRI data were acquired on a GE 3Tesla scanner (Discovery MR750, GE, Milwaukee, WI). The functional data were T2*‐weighted echo‐planar images (EPIs) consisting of 755 volumes for the stress task (details in the Supporting Information). All fMRI data preprocessing and analysis was performed in Matlab 2018a (The Mathworks Inc., Natick, MA) and SPM12 (Statistical parametric mapping software, version 12; Wellcome Department of Imaging Neuroscience, London, United Kingdom). First, data was slice‐time corrected and realigned to the first image of the task to correct for head motion. For spatial normalization, a single T2*‐weighted EPI (details in the Supporting Information) image acquired with a longer repetition time and minimum echo time was segmented using the unified segmentation scheme. While susceptibility induced signal distortions are different at different echo times, this EPI image has the same geometrical distortions as the functional images, but with higher contrast‐to‐noise ratio. The better match between this image and the fMRI volumes enables successful anatomical segmentation and non‐linear transformation to atlas space. Extracted gray matter and white matter segments were used for DARTEL (Ashburner, 2007) normalization to MNI templates. Functional images were co‐registered to the single EPI image and normalized by applying the DARTEL‐derived transformation matrix. Data was interpolated with a resolution of 2 × 2 × 2 mm. The last step was the smoothing of the data with a 6x6x6 mm FWHM kernel. During the realignment, the six head motion‐parameters were extracted for later use as nuisance covariates. Additionally, we calculated the framewise displacement for all six parameters and extracted physiological noise components based on aCompCor (Behzadi, Restom, Liau, & Liu, 2007). We extracted the voxel‐wise time series of the normalized but unsmoothed functional data from thresholded (*p* > .90) white matter and cerebrospinal fluid segments, performed PCA, and used the first five components of each segment as physiological noise covariates.

### 2.5 Data analysis

#### 2.5.1 Cortisol response to IVP

The cortisol response elicited by IVP was estimated using salivary cortisol measures. This response was calculated as the increase in salivary cortisol from T1 to T2 (∆Cort_IVP_ = Cort_T2_ − Cort_T1_). Further, we classified participants into responders and non‐responders to the IVP based on a conservative cut‐off of ∆Cort_IVP_ > 2.5 nmol/L (0.91 ng/ml, Wust et al., 2000) previously used in similar studies (Lueken, Muehlhan, Evens, Wittchen, & Kirschbaum, 2012; Muehlhan, Lueken, Wittchen, & Kirschbaum, 2011) to test if marked IVP‐induced cortisol responses alter stress task reactivity. All subsequent analyses were primarily based on the comparison between IVP responders and non‐responders, but quantitative analyses based on ∆Cort_IVP_ were also performed.

#### 2.5.2 Stress response to the psychosocial stress task

To delineate effects of psychosocial stress and IVP on cortisol concentrations over time, we quantified the HPA axis response to the task using serum cortisol measurements. We calculated the area under the curve (AUC) values starting at the beginning of the stress task and ~60 min after IVP, a time‐interval frequently recommended to aid recovery of the cortisol system. Thus, we included serum cortisol measures from the time point T3 until T8, 30 min after the end of the task. Cortisol responses may be partly offset by declining cortisol concentrations over the day starting shortly after the morning cortisol peak. Therefore, we additionally calculated cortisol concentrations corrected for a linear circadian trend between T3 and T8 and used those values to subsequently derive a circadian corrected AUC (AUC_Circ,_ for details see Supporting Information) to assess if the psychosocial stressor elicited an HPA axis response above the circadian decline. This previously used and validated (Elbau et al., 2018) approach is sensitive to small cortisol increases but does not overestimate stress effects, making it suitable to assess the success of HPA axis induction by the stress task across the whole group. However, the serum‐cortisol AUC‐values still incorporate IVP‐related effects. Moreover, serum cortisol values may not have returned to their physiological circadian level with the last available measurement. Consequently, we used the AUC values derived from uncorrected serum cortisol concentrations to assess interindividual differences induced by the IVP.

Autonomous stress effects elicited by the stress task were estimated as change in average pulse rate (beats per minute, bpm) during the arithmetic blocks in the Stress condition compared to the arithmetic blocks in the PreStress condition (∆HR_Stress_ = HR_Stress_ − HR_PreStress_). In the same way, the lasting effects of stress during the acute recovery (PostStress) phase were calculated as ∆HR_PostStress_ = HR_PostStress_ − HR_PreStress_.

Subjective stress effects elicited by the stress task were estimated as the change in positive and negative affect after the task (∆Pos/∆Neg = Positive/Negative affect (T6) − Positive/Negative affect (T3)). As in previous work (Elbau et al., 2018), we used 15 items of the BSKE to assess negative and positive affect and calculated sum scores for the relevant items (for details see Supporting Information).

The effects of cortisol induced by IVP on autonomous, subjective or serum HPA responses to the subsequent stress task were assessed with multiple linear regression models including either the responder status to IVP or the cortisol increase (∆Cort_IVP_) as predictor and sex, age, and presence of any lifetime psychiatric diagnosis (coded yes/no) as covariates.

#### 2.5.3 fMRI data

The first‐level general linear models (GLM) were built using individual onsets and durations of all task‐blocks extracted from the log files for each participant. The task was modeled with three regressors, each modeling the five arithmetic blocks (60 s) of the conditions PreStress, Stress and PostStress, respectively. In addition, we included two regressors modeling individual motor responses and verbal feedback during the Stress phase. Nuisance regressors were the six movement parameters derived from realignment, their derivatives, and five physiological noise components extracted from white matter and cerebrospinal fluid each. Data were high pass filtered with a cut‐off of 256 s. The contrasts of interest, *Stress–PreStress*, to assess acute psychsocial stress, and *PostStress–PreStress*, to assess effects of fast stress recovery, were estimated for each participant. To additionally describe effects on a network level, we aggregated stress effects by calculating mean betas within networks (Yeo et al., 2011) for each participant and contrast and tested for significant changes within one network across participants.

To test the effects of IVP‐induced cortisol responses on the subsequent neural stress response to the psychosocial stressor, we performed whole‐brain voxel‐wise multiple regression analyses using the contrast images derived in the first‐level statistics. Either ∆Cort_IVP_ or responder status were included as predictor and sex, age, and lifetime psychiatric diagnosis (no/yes) as covariates. In addition to this cluster‐based approach, we again used aggregated betas within networks to compare neural stress responses in IVP‐responders and non‐responders. We depict *t* values (i.e., a ratio of the beta coefficients and their variability) in Figure 3 to ease their comparison.

However, this approach may mask individual variation in the direction or localization of stress effects. To capture individual neural stress effects independent of their directionality and localization, we calculated within‐participant similarity of the neural activity during PreStress compared to Stress and PostStress, respectively. To this end, we extracted mean beta estimates of the conditions (PreStress, Stress, and PostStress) from 268 regions of interest (ROIs) spanning the whole brain using an established brain parcellation (Shen, Tokoglu, Papademetris, & Constable, 2013) to assess stress effects on the ROI level. Representational similarity was then calculated as the Pearson correlation between the activity PreStress and Stress or PressStress and PostStress across all ROIs for each participant separately. In addition, we used voxel‐wise beta coefficients and estimated individual similarity within functional Yeo networks (Yeo et al., 2011) to test an alternative level of aggregation. Correlation coefficients were Fisher's z‐transformed for further parametric analyses. Effects of IVP‐induced cortisol on neural similarity during and after stress were tested by applying linear models including sex, age, lifetime diagnosis, and average framewise displacement as covariates.

#### 2.5.4 Statistical threshold and software

Statistical analyses were performed in R v3.5.1. (R Core Team, 2018). To account for non‐normal distributions of the cortisol responses, we additionally bootstrapped all regression estimates (2,000 resamples). As the current literature did not converge to suggest a heightened or attenuated stress response after IVP, we used two‐sided tests with a significance threshold *p* < .05 for all effects of interest. For whole‐brain fMRI analyses, the voxel threshold was set at *p* < .001 (uncorrected). Clusters were considered as significant with an FWE cluster‐corrected *p* value threshold of *p*_cluster.FWE_ < .05.

## 3 RESULTS

### 3.1 The imaging stress task induced autonomous, subjective, and neural stress responses

**FIGURE 2:**
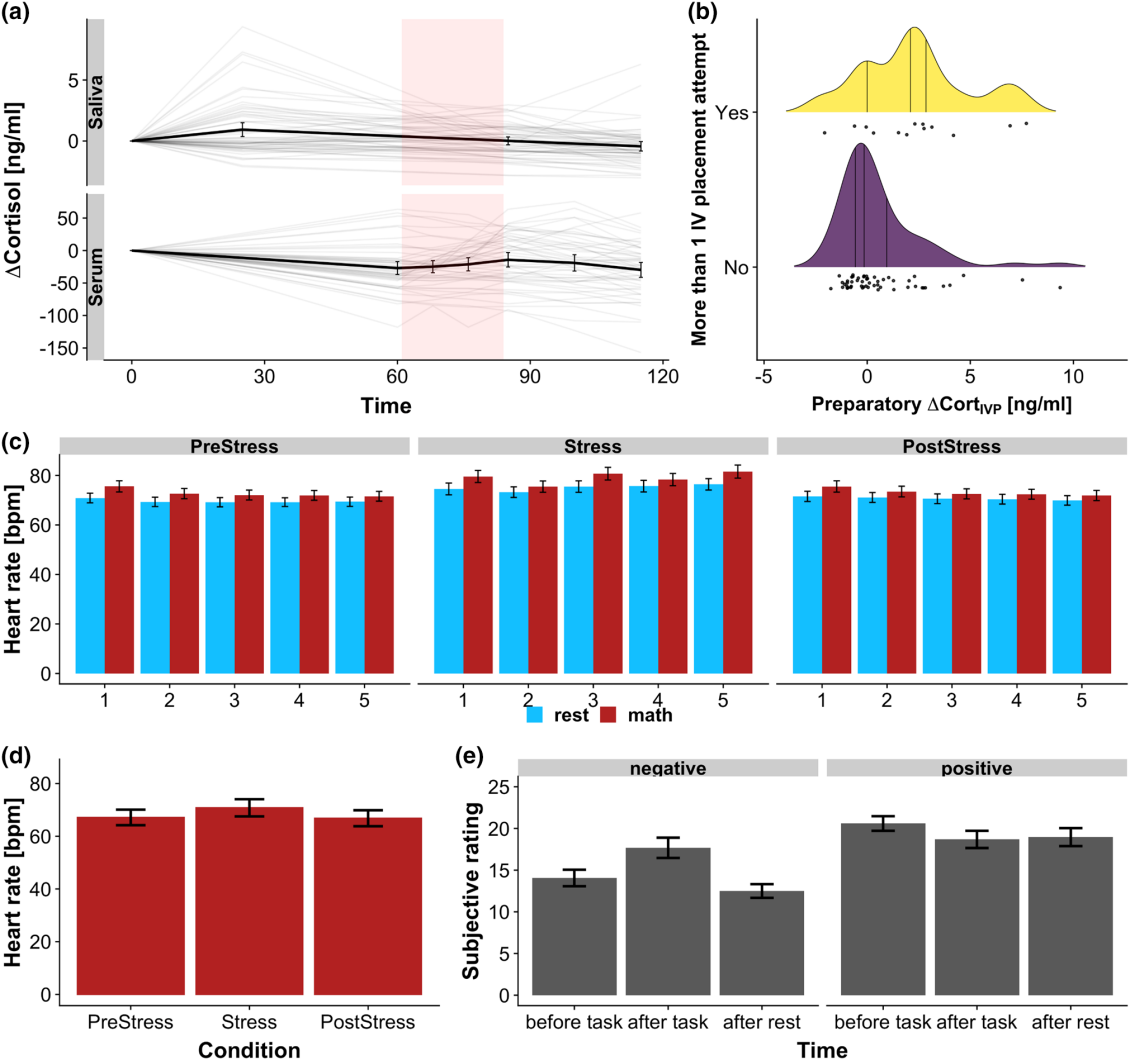
The intravenous catheter placement (IVP) and the stress task increased stress levels. (a) Cortisol response (∆Cortisol) over time. IVP just before T1 increased average salivary cortisol at T2. Thin lines depict individual cortisol profiles, thick lines depict the group average. Shaded rectangles indicate the stress task phase. (b) IVP‐induced cortisol responses were higher after complicated placement, for example, if more than one attempt was needed. (c) The average pulse rate was higher during the cognitive task (math) compared to rest phases across all task blocks. (d) The average pulse rate in math phases of the Stress condition was higher compared to PreStress and PostStress. Notably, the average pulse rate did not completely recover. (e) Stress increased negative emotions and decreased positive emotions. Increases in negative emotions were transient and recovered back to baseline levels, while positive emotions remained reduced. Error bars depict 95% confidence intervals

First, we assessed if stress induction by the imaging stress task was successful. As expected, stress induction increased the pulse rate in the Stress (mean ∆HR_Stress_ = 8.17 bpm, *t*[55] = 9.34, *p* < .0001) and PostStress (mean ∆HR_PostStress_ = 1.42 bpm, *t*[55] = 2.11, *p* = .038) phase. Still, it recovered significantly (mean ∆HR_Stress‐PostStress_ = −6.74 bpm, *t*[55] = 8.39, *p* < .0001) after stress (see Figure 2c,d). Positive affect was decreased (mean ∆Pos(T6) = −1.81, *t*(66) = −3.30, *p* = .0002) and negative affect increased (mean ∆Neg(T6) = 6.16, *t*(66) = 6.48, *p* < .0001) directly after the task (Figure 2e). In contrast, only positive affect was still decreased (mean ∆Pos(T8) = −1.13, *t*(66) = −2.16, *p* = .034) 30 min later while negative affect had recovered to levels slightly below baseline (mean ∆Neg(T8) = −0.82, *t*(66) = −1.26, *p* = .21, Figure 2e). Both, pulse rate increases in the Stress and PostStress phase as well as affect changes directly and 30 min after the task, were positively correlated (*r*s between 0.63 and 0.77, all *ps* < .0001) indicating that interindividual differences of the autonomous and subjective stress response also persist during the recovery period. The task elicited a significant serum cortisol response (AUC_Circ_ = 459 ng/ml × min, *t*[51] = 3.69, *p* = .0005) when taking into account the approximated circadian cortisol decline for each individual. Eighteen participants would be classified as responders with a peak cortisol response higher than 55 nmol/L (19.99 ng/ml, equivalent to 2.5 nmol/L threshold in saliva).

**FIGURE 3:**
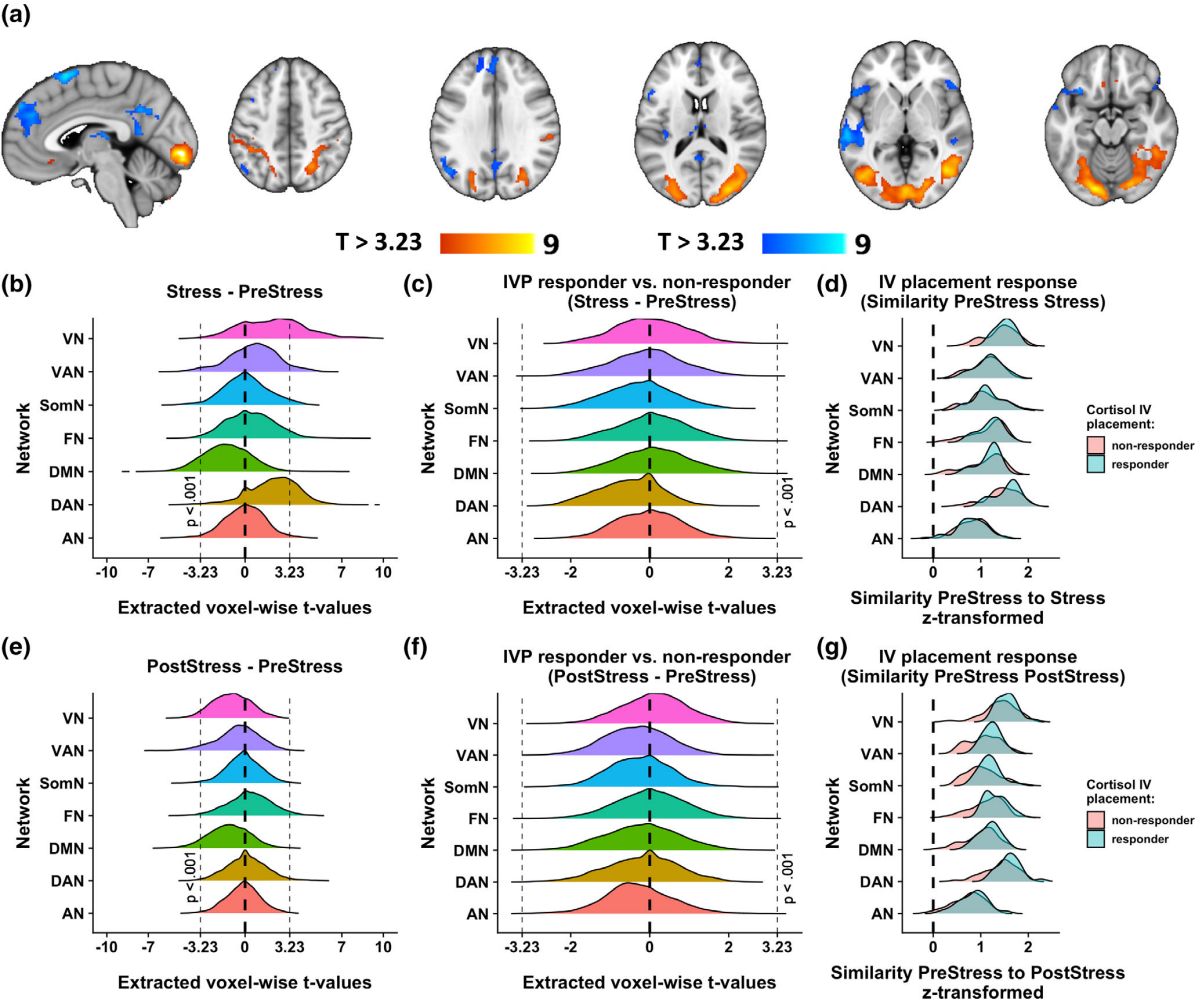
Changes in stress‐induced brain activity depend on the IVP cortisol response. (a) Stress‐induced (Stress–PreStress) activation (warm colors) and deactivation (cool colors) across all participants, voxel‐threshold *p* < .001. (b) Activity during stress was reduced in the default mode network (*t*(66) = −3.24, *p* = .0018) and increased in the dorsal attention (*t*(66) = 5.11, *p* < .0001) and visual network (*t*(66) = 4.9, *p* < .0001). (c) No network‐specific differences comparing IVP responders to non‐responders in the contrast Stress–PreStress. Less than 100 voxels exceeded the *t* value threshold corresponding to *p*_voxel_._uncorrected_ < .001 (*t* = 3.23) and no clusters reached significance in whole brain analyses. (d) Intraindividual similarity (z‐transformed Pearson correlation) between voxel‐wise neural activity during Stress compared to PreStress was not different in IVP responders. (e) Activity after stress remained reduced in the default mode network (*t*(66) = −3.9, *p* = .00017) but recovered in all other networks. (f) IVP responders and non‐responders did not differ in network‐specific activity in the contrast PostStress–PreStress. Less than 100 voxels exceeded the *t* value threshold corresponding to *p*_voxel_._uncorrected_ < .001 (*t* = 3.23) and no clusters reached significance in whole brain analyses. (g) Intraindividual similarity (z‐transformed Pearson correlation) between voxel‐wise neural activity during PostStress compared to PreStress was higher in IVP responders across functional networks. (b)–(g) depict the density of voxel‐wise extracted *t* values for the following functional networks: VN, visual network; VAN, ventral attention network; SomS, somatosensory network; FN, frontoparietal network; DMN, default mode network; DAN, dorsal attention network; AN, limbic network

Likewise, stress‐induced changes in neural activity, as assessed within the contrast PreStress‐Stress, mapped to increased activity in primary and secondary visual as well as lateral parietal cortex and decreased activity in the default mode network, including the posterior cingulate cortex (PCC), precuneus and lateral parietal (angular gyrus) and temporal cortex, dorsomedial prefrontal cortex, thalamus, and insula (Figure 3a). Consistent with this voxel‐wise approach, at the network level, increases in activity were predominantly observed in the visual and the dorsal attention network (Yeo et al., 2011), while deactivation were observed in the default mode network (Figure 3b). Moreover, the deactivation of the default mode network was still visible in the PostStress phase, while activation of the dorsal attention network recovered closer back to baseline (Figure 3e).

### 3.2 IVP increased salivary cortisol

The placement of the IV led to a significant salivary cortisol response 20 min later (T1, mean ∆Cort_IVP_ = 0.93 ng/ml, SD ∆Cort_IVP_ = 2.29 ng/ml, *t*[64] = 3.27, *p* = .001, *p*_boot_ < .001, Figure 2; raw cortisol concentrations Figure S1). Importantly, 35.4% (*n* = 23) of the participants reacted to IVP with a cortisol response larger than 2.5 nmol/L (0.91 ng/ml, Wust et al., 2000), indicating substantial interindividual differences. Differences in cortisol response to IVP were not dependent on baseline cortisol concentrations (serum: *t*(52) = −0.18, *p* = .85; saliva: *t*(63) = −0.03, *p* = .98). Serum and salivary cortisol at baseline were highly correlated (*r* = .61, *p* < .0001). Of note, cortisol responses to IVP were higher in participants for whom more than one attempt was needed until success (*b* = 1.85 ng/ml, *t*[60] = 2.65, *p* = .010, Figure 2b). In contrast, reporting at least one symptom of needle phobia was not predictive of the cortisol response to IVP (*b* = −1.13 ng/ml, *t*[60] = −1.11, *p* = .27). Responders to IVP did not differ from non‐responders with respect to various other demographic and psychopathological variables (Table 1).

**TABLE 1:** Sociodemographic and psychopathological information of IVP responders and non‐responders

	IVP‐responder *N* = 23	IVP non‐responder *N* = 42	Statistic (*χ* ^2^ or *t* value)	*p*
Age	32.78 ± 8.71	31.52 ± 9.97	−0.53	.60
Sex: Female	7	18	0.52	.47
Problems IVP	9	4	8.1	.004**
At least one symptom of needle phobia	1	5	1.01	.31
Depression‐related diagnosis (F3)				
12 months	0	4	2.22	.14
Lifetime	1	4	0.49	.48
Anxiety‐related diagnosis (F4)				
12 months	2	10	2.02	.15
Lifetime	7	19	1.42	.23
Substance abuse disorders (F1)				
12 months	2	1	1.47	.23
Lifetime	3	10	0.91	.34
Other psychiatric disorders				
12 months	0	0	n.a	n.a.
Lifetime	3	2	1.46	.23
Any lifetime psychiatric disorder: Yes	9	25	2.48	.12

*Note:* Diagnoses are derived from the automatically evaluated CIDI‐interview.

Abbreviation: IVP, intravenous catheter placement.

** *p* < .01.

### 3.3 The endocrine response to psychosocial stress was lower in IVP‐responders

**FIGURE 4:**
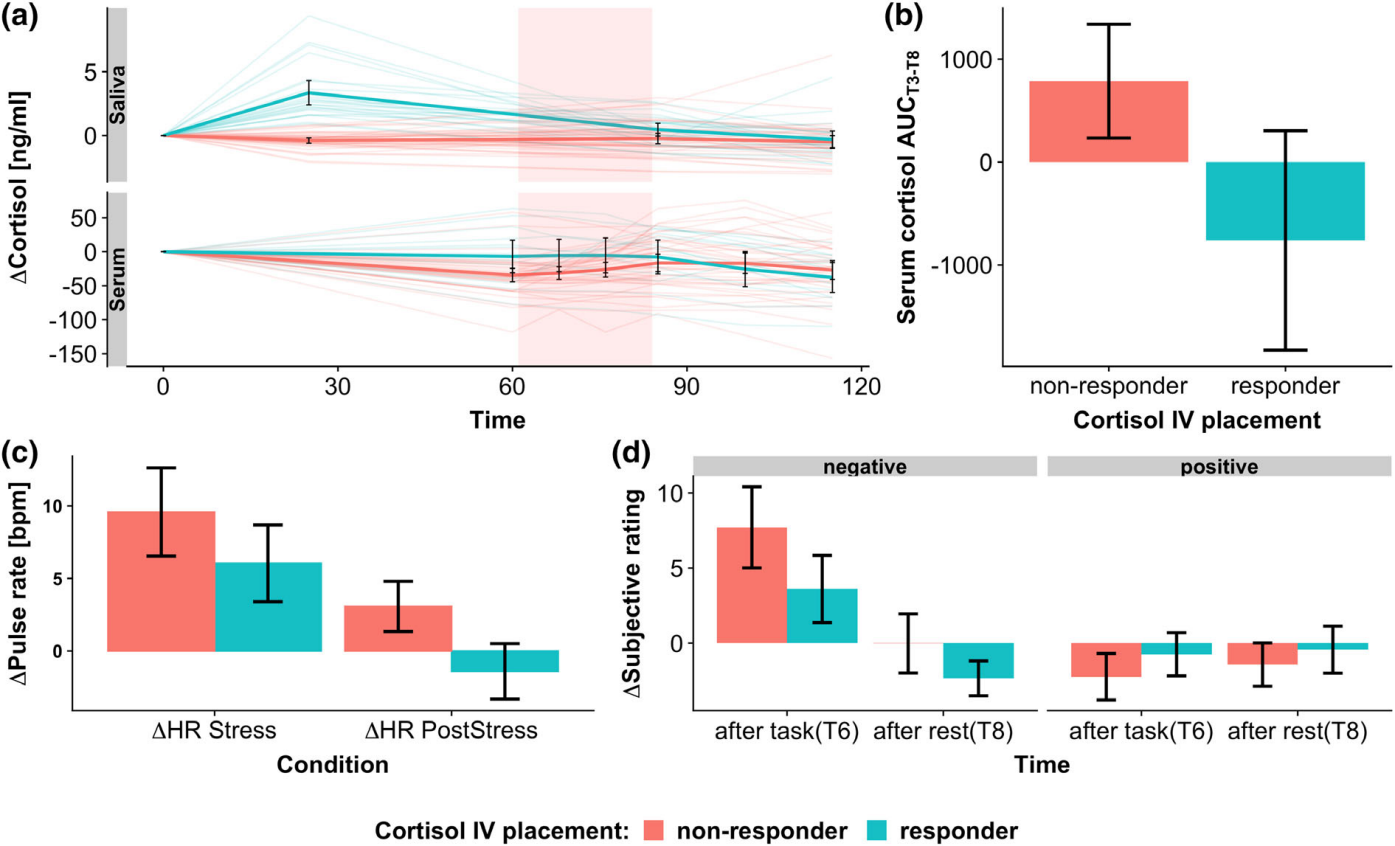
Response to the stress task depends on IVP response. Cortisol responders (∆Cort_IVP_ > 2.5 nmol/L, [Wust et al., 2000]) to the intravenous catheter placement (IVP) show reduced endocrine, autonomous, and subjective reactivity to the stress task. (a) Cortisol response (∆Cortisol) over time. IV placement before T1 increases salivary cortisol at T2. Note that serum cortisol values were still slightly elevated in responders compared to non‐responder even 60 min after IVP. Thin lines depict individual cortisol profiles, thick lines depict the mean cortisol response in IVP responders/non‐responders. Shaded rectangles indicate the task phase. (b) Serum cortisol response to the stress task (AUC_T3‐T8_) is reduced in IVP responders compared to non‐responders. (c) The pulse rate response to the stress task is reduced in cortisol responders to IVP, especially in the PostStress phase. (d) Increase in negative emotions is reduced in non‐responders compared to responders. Positive affect is unaffected. Error bars depict 95% confidence intervals

To investigate the effects of IVP on the cortisol response trajectories, we tested if a strong response to the IVP alters stress task reactivity. The endocrine response to the stress task (AUC_serum.T3‐T8_) was lower in IVP responders compared to non‐responders (difference = −1,459 ng/ml × min, *p* = .013, *CI*_boot_ = [−2,584 ng/ml × min to −264 ng/ml × min], Figure 4b). Critically, serum cortisol levels before the start of the task were lower than at baseline (*mean* ∆SerumCort_T3_ = −27.3 ng/ml, *t*[53] = −5.75, *p* < .001, Figure 4a) across the whole sample, but changes of serum cortisol between baseline and the start of the task (T3) were dependent on the cortisol response to IVP with responders having significantly higher cortisol changes than non‐responders (*t*[50] = −2.78 ng/ml, *p* = .008, correlation ∆Cort_IVP_: *r* = .41, *p* = .002, Figure 4a). Collectively, this indicates that IVP alters the cortisol system for at least the following 60 min potentially influencing the cortisol response to the subsequent stress task.

### 3.4 Reduced stress reactivity and facilitated recovery in IVP responders

The reduced endocrine response in IVP responders was mirrored in a reduced autonomous and subjective response predominantly in the PostStress recovery phase. Here, lasting pulse rate increases during PostStress compared to PreStress were 4.9 bpm (*n* = 54, *p* = .0006, *CI*_boot_ = [−7.39 to −2.65]) lower in IVP responders (see Figure 4c). Likewise, increases in negative affect directly after the PostStress condition of the task were reduced by 55% (*b* = −4.47, *p* = .034, *CI*_boot_ = [−8.15 to −0.82]) in IVP responders compared with non‐responders. In contrast, positive affect and pulse rate increases during stress were not significantly different in responders compared to non‐responders (Figure 4d, Table 2). Interestingly, IVP responders had a significantly higher pulse rate already in the PreStress phase (*b* = 8.30, *p* = .020, *CI*_boot_ = [0.84–15.13]). In contrast, there were no differences in the affective state (positive: *b* = 0.23, *p* = .81, *CI*_boot_ = [−1.42 to 1.80], negative: *b* = 0.51, *p* = .67, *CI*_boot_ = [−1.66 to 2.64]) directly before the task (T3). Comparable results were obtained when using the quantitative salivary cortisol response to IVP as a predictor (Table 2, Figure S3).

**TABLE 2:** Cortisol response to IVP influenced the stress response to the subsequent psychosocial stress task

	∆Cort_IV_			IVP reponder/non‐responder		
	*b*	*p*	*CI*_boot_	*b*	*p*	*CI*_boot_
*Cortisol (N = 54)*						
AUC (ng/ml × min)	−245	.049	[−487 to −40]	−1,459	.013	[−2,584 to −264]
*Pulse rate (N = 54)*						
PreStress (bpm)	1.27	.087	[0.03 to 3.05]	8.30	.020	[0.85 to 15.12]
∆Stress (bpm)	−0.71	.13	[−1.79 to 0.01]	−4.45	.050	[−8.72 to −0.53]
∆PostStress (bpm)	−0.64	.035	[−1.51 to −0.18]	−4.93	.0006	[−7.39 to −2.64]
*Subjective (N = 65)*						
Positive PreTask	0.11	.58	[−0.24 to 0.38]	0.23	.81	[−1.41 to 1.80]
Negative PreTask	0.23	.34	[−0.27 to 0.67]	0.51	.67	[−1.67 to 2.64]
∆Positive	0.25	.32	[− 0.15 to 0.84]	1.72	.15	[−0.39 to 4.09]
∆Negative	−0.89	.043	[−1.83 to −0.13]	−4.47	.034	[−8.15 to −0.82]
*Neural (ROI) (N = 65)*						
Similarity pre‐stress	0.02	.19	[−0.01 to 0.04]	0.09	.22	[−0.06 to 0.22]
Similarity pre‐post	0.03	.049	[0.002 to 0.05]	0.16	.024	[0.02 to 0.30]

*Note*: Regression weights from linear models including the different cortisol measures as predictor and age, sex, lifetime‐diagnosis status (and framewise displacement for neural similarities) as covariates.

Abbreviations: CI, bootstrapped 95% confidence intervals; IVP, intravenous catheter placement.

In contrast, region‐specific, neural activity before stress (PreStress), during stress (Stress–PreStress) or recovery (PostStress–PreStress) was not different in IVP responders compared to non‐responders, as whole‐brain voxel‐wise analysis revealed no significantly different clusters even without further correction for multiple comparisons. Moreover, additional network‐level analysis showed that there were no low‐intensity shifts in activity in any of the main functional networks (Yeo et al., 2011) (Figure 3c,f). Nevertheless, neural similarity between PostStress and PreStress neural activity, a measure capturing stress‐induced changes that are not necessarily region‐specific or in the same direction between individuals, was higher in IVP responders. We assessed similarity of stress responses using either an aggregation at the network or one at the ROI level. In both analyses, similarity was significantly higher in IVP responders (ROI: *b* = 0.23, *p* = .003, *CI*_boot_ = [0.10–0.35] Figure S2; Network: *b* = 0.06, *p* = .028, Figure 3g). Following correction for the described nuisance effects (age, sex, diagnosis, average FD), the difference remained significant at the ROI level but not for the network aggregation (ROI: *b* = 0.16, *p* = .024, *CI*_boot_ = [0.02–0.30] Figure S2; Network: *b* = 0.04, *p* = .127, Figure 3g) suggesting that the latter analysis was more affected by confounds. Collectively, the results suggest faster recovery to baseline levels in IVP responders, perhaps due to the earlier trigger of the HPA axis response.

## 4 DISCUSSION

Stress reactivity is often quantified using validated and standardized procedures (Allen et al., 2014; Kirschbaum et al., 1993) as reliable quantification within as well as between individuals of stress reactivity is crucial for the identification of response profiles predictive of psychopathological risk. Nonetheless, there are numerous variations of protocols across studies and even slight modifications may elicit a preceding cortisol response that alters the baseline state of the HPA axis and thereby influences the individual response to the main experimental stressor (Goodman et al., 2017). One frequent protocol modification is the placement of an IV to measure serum cortisol across time. Here, we investigated if individual differences in cortisol responses to IVP are associated with altered reactivity to a subsequent psychosocial stress task. IVP elicited a relevant cortisol response in over 30% of the sample. Moreover, in those participants, cortisol levels remained elevated up to the start of the stress task and a blunted cortisol response was elicited by the task. This was paralleled on the autonomous, neural, and subjective level, which all showed less reactivity to the task or faster return to baseline in IVP responders. This is in line with previous observations that stress reactivity is reduced in case of higher baseline cortisol (Dickerson & Kemeny, 2004; Kudielka et al., 2004). Task‐unrelated prior cortisol responses may thus limit the individual response to a subsequent psychosocial stressor and confound inter‐individual differences in stress reactivity.

Our finding that pre‐task IVP‐induced cortisol increases reduced the endocrine response to a subsequent psychosocial stress task could be explained by habituation of the HPA axis. This has previously been described after repeated participation in a stress task on the same day, indicating the possibility of desensitization of the HPA axis within a certain time window of repeated stimulation (Höhne et al., 2014). Importantly, habituation may extend to stressors unrelated to the stress task such as physical exercise (Zschucke et al., 2015). The attenuating effects of a first cortisol response could be related to glucocorticoid receptor (GR)‐mediated negative feedback on the HPA axis at the level of the pituitary and the brain (Herman, Ostrander, Mueller, & Figueiredo, 2005; Ulrich‐Lai & Herman, 2009) that would counteract the response to a second stimulus.

The attenuated endocrine response to the task was mirrored by reduced autonomous, psychological, and neural stress reactivity which may arise for different reasons. For instance, the IVP‐induced cortisol response could attenuate a second cortisol response and this, in turn, could translate to lower autonomous, neural, and psychological responses. Exogenous administration of steroids has been associated with acute increases in heart rate and decreased heart rate variability (Adlan et al., 2018; Dodt, Keyser, Mölle, Fehm, & Elam, 2000) and changes in neural activity of the hippocampus (Symonds, McKie, Elliott, William Deakin, & Anderson, 2012). While a previous study showed that cortisol increases self‐reported arousal (Abercrombie, Kalin, & Davidson, 2005), there is little evidence for acute cortisol effects on mood (Putman & Roelofs, 2011). Still, cortisol predominantly improved mood in response to subsequent stress challenges (Het & Wolf, 2007; Soravia et al., 2006), comparable to the attenuated negative emotional response to the stress task in our study. Therefore, reduced stress reactivity across response systems as observed in our study could also reflect the previously proposed restorative role of delayed GR‐mediated processes (de Kloet, Joëls, & Holsboer, 2005) elicited by the cortisol response to the IVP.

Alternatively, the preceding HPA axis response to IVP could have induced persistent changes in heart rate or mood that subsequently tune the response to the psychosocial stress task. Stress responses are initiated by brain circuits that integrate psychological information, such as salience, valence, and context on the stressor with current homeostatic information (Ulrich‐Lai & Herman, 2009). For example, homeostatic indices of resting autonomous functioning have been shown to predict the cortisol response to a stress task (Weber et al., 2010). Comparably, IVP responders showed an increased task‐associated pulse rate in the PreStress phase, indicating potentially lasting effects of the pre‐task stress response. This was accompanied by an attenuated response to the psychosocial stressor suggesting limited excitability to subsequent stimuli. Lasting changes in mood, context, or expectations about the following task could also influence the response to the stress task (Salzmann et al., 2018). However, IVP‐responders did not differ in their self‐reported mood directly before the stress task. Nonetheless, the cognitive appraisal of physiological responses is crucial for the generation of the emotional response (Folkman et al., 1986; Ursin & Eriksen, 2004) and is also influenced by pre‐stress expectations and other cognitive strategies (Gaab, Rohleder, Nater, & Ehlert, 2005; Jamieson, Hangen, Lee, & Yeager, 2018). Thus, the attenuated negative response to the stress task in IVP responders may suggest that any additional physiological response induced by the psychosocial stressor was perceived as less aversive than the relief of physiological stress from the IVP‐induced response leading to an attenuated negative appraisal.

Contrary to our hypothesis, cortisol responses induced by IVP did not reduce baseline activity or neural stress reactivity in specific brain regions, specific clusters in whole‐brain voxel‐wise analysis or even on a broader network level. Critically, the psychosocial stressor in the task induced the expected increase in activity in the dorsal attention and visual networks and stronger deactivation in the DMN (Dedovic, D'Aguiar, & Pruessner, 2009; Elbau et al., 2018). Interestingly, the DMN still maintained a stronger deactivation in the PostStress task phase, which is in line with previously reported changes in connectivity of the default mode network up to 2 hr after stress induction (Veer et al., 2011; Zhang et al., 2019). However, those stress‐induced changes of activity in the dorsal attention, visual, and default mode network did not differ between IVP responders and non‐responders. Likewise, yet in contrast to previous reports (Zschucke et al., 2015), we did not observe any differentially activated clusters in participants showing an IVP cortisol response. One explanation for the diverging results could be the high heterogeneity of imaging stress studies with regards to specific procedures, leading to variable group‐level stress effects and little convergence (Kogler et al., 2015; Noack et al., 2019). Likewise, neural effects may be masked by high inter‐individual variability of the localization and maybe even directionality of the neural stress effects. Recently, representational similarity analysis has been used to re‐identify participants with high accuracy across different tasks analogous to “fingerprinting” (Finn et al., 2015). Due to the high reliability of individual connectomes or specific task‐induced brain activation patterns (Fröhner, Teckentrup, Smolka, & Kroemer, 2019), representational similarity can be used to track changes from an individual baseline regardless of the direction. Indeed, within‐participant similarity between the PreStress and PostStress condition was higher in IVP responders suggesting faster recovery back to PreStress neural activity.

Differences between IVP responders and non‐responders were predominantly observed in the PostStress phase for autonomous as well as neural responses, while acute changes under stress were less affected. Comparably, pre‐task exercise stress did not alter acute HR increases (Hamer, Taylor, & Steptoe, 2006) to a subsequent stressor, but changes in stress recovery after pre‐treatment with cortisol have been reported (Soravia et al., 2006). One explanation may be stronger influence of high‐level interindividual differences in moderating factors such as coping or resilience on post stress recovery (Lü, Wang, & You, 2016). Likewise, preservative cognitions or extended rumination after stress have been related to longer lasting physiological alterations after stress and are likely also supported by lasting alterations in neural activity (Brosschot, Gerin, & Thayer, 2006; Ottaviani et al., 2016). Nonetheless, specificity of effects for the PostStress phase is limited, as the responses in both conditions were highly correlated.

This study has several limitations. First, we assessed the impact of an IVP‐induced cortisol response on a subsequent stress response within participants. This is necessary to capture the individual variability in the response to IVP and determine effects of a preceding cortisol response on the subsequent stress response. However, we did not include a control condition where the same participants or a control group took part in the stress task without prior IVP. Therefore, future studies are necessary to confirm that stress reducing effects of pre‐task IVP are only present in responders. Also, we cannot identify which exact factor of the IVP procedure caused the HPA axis response (needle phobia, painful procedure). Nonetheless, the effects of any adaptations to the procedure that could induce a pre‐task cortisol response, should be assessed by repeated cortisol measurements even before the start of the task. Second, we only assessed the cortisol response to IVP and did not concurrently measure pulse rate or the subjective experience of blood taking. However, this information may help to understand how the appraisal of the different physiological responses influences subsequent stress reactivity (Gaab et al., 2005). Third, other parts of the procedure, for example, anticipation of the MR environment, may also lead to additional inter‐individually different perturbations of the stress system that consequently alter stress reactivity (Muehlhan et al., 2011). However, none of the participants were MRI‐naïve, reducing potential confounding by individual differences in previous MRI‐exposure. Fourth, a number of other confounding factors, such as hormonal status, use of contraceptives and importantly time of day may also influence results. As all sessions started at around 10 a.m., we cannot generalize our results to other times of the day beyond the afternoon, when cortisol baseline levels are lower.

In summary, the IVP led to a significant cortisol response in 35% of the participants. Critically, in these IVP responders, reactivity to the psychosocial stress task was significantly reduced including lower endocrine, subjective, autonomous, and neural responses. These effects were found despite a delay of about 60 min between IVP and the start of the stress task, a time frame that has often been considered as sufficient to avoid carry‐over effects. Collectively, our results suggest that an unrelated cortisol response that is induced before a psychological stressor may have beneficial, stress‐reducing effects in a consecutive stressful situation. Interestingly, this is not only the case for “positive” stressors such as exercise (Zschucke et al., 2015), but also for presumably aversive stressors such as placement of an IV. Moreover, the study further emphasizes the importance of high frequency cortisol assessment in stress studies to identify sources of individual variability in responsivity. Thus, high heterogeneity in the specifics of measurements (mode, frequency, invasiveness) and interindividual differences in the response to different parts of the procedures may reduce the meta‐analytic convergence across studies calling for a stronger emphasis on standardization of procedures and replicability.

## CONFLICT OF INTEREST

The authors declare no potential conflict of interest.

## AUTHOR CONTRIBUTIONS

**Elisabeth B. Binder** and **Philipp G. Sämann:** were responsible for the study concept and design**. Immanuel G. Elbau**, **Michael Czisch** and **Philipp G. Sämann:** validated the paradigm and procedure. **Anne Kühnel:** performed the data analysis and **Nils B. Kroemer:** contributed to analyses. **Anne Kühnel:** wrote the manuscript. All authors contributed to the interpretation of findings, provided critical revision of the manuscript for important intellectual content and approved the final version for publication.

## Supporting information


**Appendix** **S1.** Supporting Information.

## Data Availability

The data that support the findings of this study are available from the corresponding author upon reasonable request.
