# Supplementary material for: Psychosocial stress reactivity habituates following acute physiological stress
Source: Hum Brain Mapp. 2020 Jun 29;41(14):4010–23. doi: 10.1002/hbm.25106 (PMC7469805; doi:10.1002/hbm.25106)
Supplement: Supplementary file 1 — Appendix S1. Supporting Information. [file HBM-41-4010-s001.docx]

# **Supporting Information:**

**Psychosocial stress reactivity habituates following acute physiological stress**

Anne Kühnel^1,2*^, Nils B. Kroemer^3^, Immanuel G. Elbau^1^,

Michael Czisch^4^, Philipp G. Sämann^4^, Martin Walter^3,5-7^,

BeCOME working group^1,4^ & Elisabeth B. Binder^1*^

^1^ Department of Translational Research in Psychiatry, Max Planck Institute of Psychiatry

^2^ International Max Planck Research School for Translational Psychiatry (IMPRS-TP), Munich, Germany

^3^ Department of Psychiatry and Psychotherapy, University of Tübingen, Tübingen, Germany

^4^ Max Planck Institute of Psychiatry, Munich, Germany

^5^ Leibniz Institute for Neurobiology, Magdeburg, Germany

^6^ Otto-von-Guericke University Magdeburg, Department of Psychiatry and Psychotherapy, Magdeburg, Germany

^7^ Department of Psychiatry and Psychotherapy, Jena University Hospital, Jena, Germany

**Corresponding authors***

A. Kühnel, anne_kuehnel@psych.mpg.de

E. Binder, binder@psych.mpg.de

BeCome working group: Tanja M. Brückl^1^, Victor I. Spoormaker^1^, Angelika Erhardt^1^, Norma C. Grandi^1^, Sanja Ilic-Cocic^4^, Susanne Lucae^4^, Alina Tontsch^1^

## **Assessment of subjective stress experience (BSKE scales)**

The BSKE (Befindlichkeitsskalierung durch Kategorien und Eigenschaftswörter, Janke, 1994) scales are a short version of the more extensive Eigenschaftswörterliste (EWL, (Janke and Debus, 1978) developed to assess the current emotional state across positive and negative dimensions. The scale consists of 15 items (emotions / states) and participants were asked to rate their current state/feeling (“I feel …”) on 6-point scale ranging from 1 (“not at all / gar nicht”) to 6 (“very strongly / sehr stark”). We calculated sum scores including the items activity, wakefulness, self-certainty, focus, and relaxed state of mind for positive affect and including the items internal and external agitation, anxiety, sadness, anger, dysphoria, sensitivity as well as three items assessing somatic changes for negative affect.

Janke, W., 1994. Befindlichkeitsskalierung durch Kategorien und Eigenschaftswörter: BSKE (EWL) nach Janke, Debus, Erdmann und Hüppe. Test und Handanweisung. Unveröffentlichter Institutsbericht, Lehrstuhl für Biologische und Klinische Psychologie der Universität Würzburg.

Janke, W., Debus, G., 1978. Die Eigenschaftswörterliste: EWL. Verlag für Psychologie CJ Hogrefe.

**fMRI Imaging parameters:**

The following scanner settings were used for acquisition of echo-planar images (EPI) for the imaging stress task: 42 oblique slices, oriented along the AC-PC plane, covering the whole brain, interleaved ascending acquisition order, TR= 2s, TE= 40ms, 64 × 64 matrix, field of view = 200 × 200 mm, voxel size = 3.5 × 3.5 × 3 mm. Additionally, the measurements included single EPI (T2*-weighted) volume with the same settings as the fMRI sequence, but a longer TR of 10s. This single EPI volume has the same geometric distortions as the fMRI images combined with a higher contrast-to-noise ratio and less signal drop-out and was used for segmentation and subsequent normalization to correct for field distortions.

**Calculation of Circadian corrected AUC values:**

In short, an individual linear baseline over time was calculated from the e cortisol assessment before the start of the stress task (T3) and the last measurement approximately 20 minutes after the end of the stress task (T8): ${Cortisol}_{circ}\left( T \right)= \frac{Cortisol \left( T8 \right)-Cortisol \left( T3 \right)}{T8}*T+Cortisol (T3)$, where T is any time starting from the onset of the stress task (T3) and T3 and T8 are the times of the 3rd and 8th cortisol measurement respectively.

The corrected cortisol response for each timepoint was then determined as a difference between the measured cortisol concentration at each timepoint and the projected baseline cortisol level at that time: ${Cortisol}_{circ.cor}=Cortisol \left( T \right)-{Cortisol}_{circ} (T)$. Lastly, we calculated the area under the curve using the corrected cortisol concentrations for the timepoints T3 until T8 and the appropriate individual time intervals.

## **Figures**


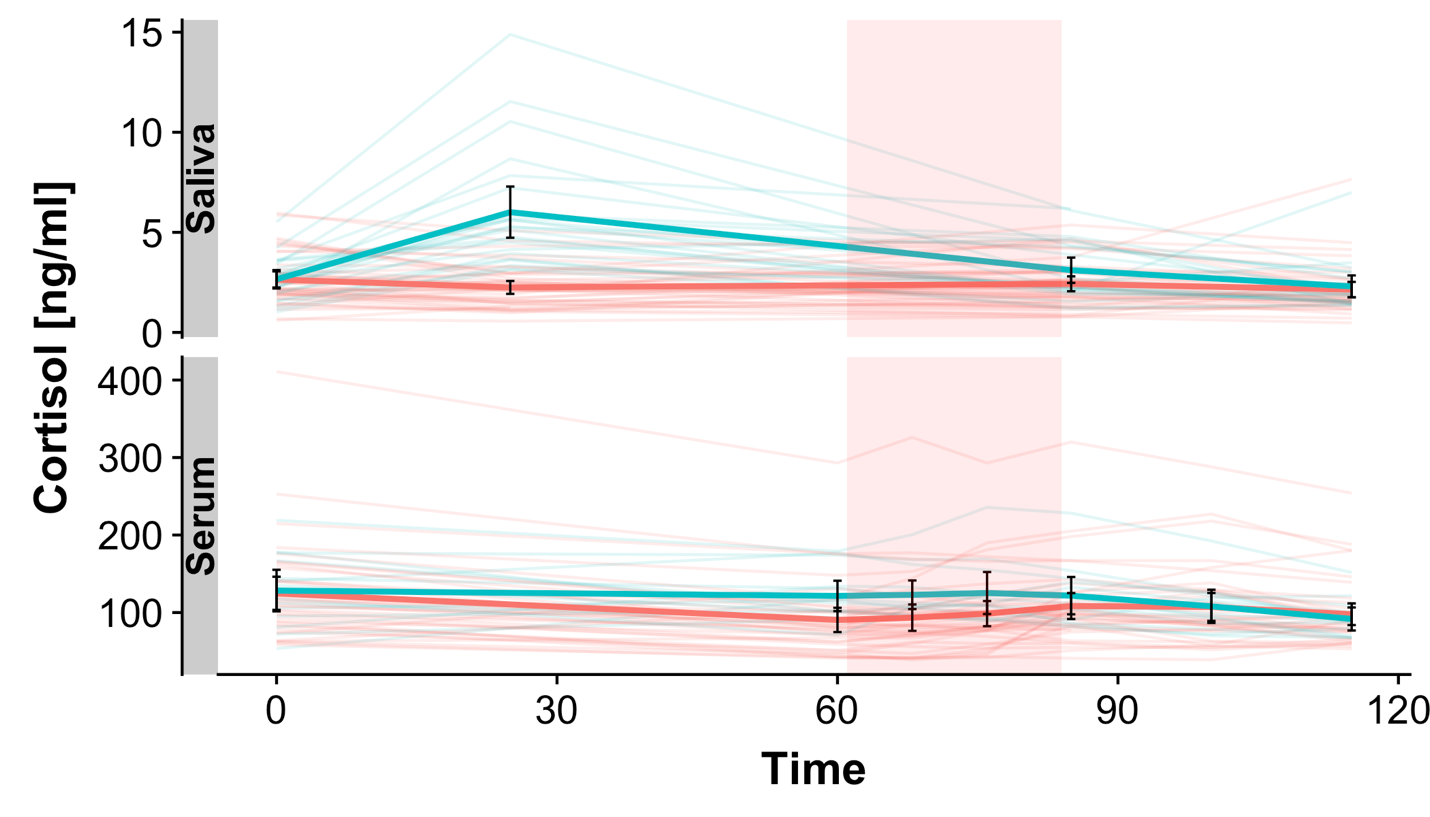


**Figure S1:** Cortisol concentrations in blood and saliva across time. Cortisol response (∆Cortisol) over time. IV placement before T1 increases salivary cortisol at T2. Note that serum cortisol values were still slightly elevated in responders compared to non-responder even 60 minutes after IVP. Red depicts non-responders, blue responders (∆CortIVP > 2.5 nmol/l / 0.91 ng/mL) to IV placement. Thin lines depict individual cortisol profiles, thick lines depict the mean cortisol response in IVP-responders/non-responders. Shaded rectangles indicate the task phase.


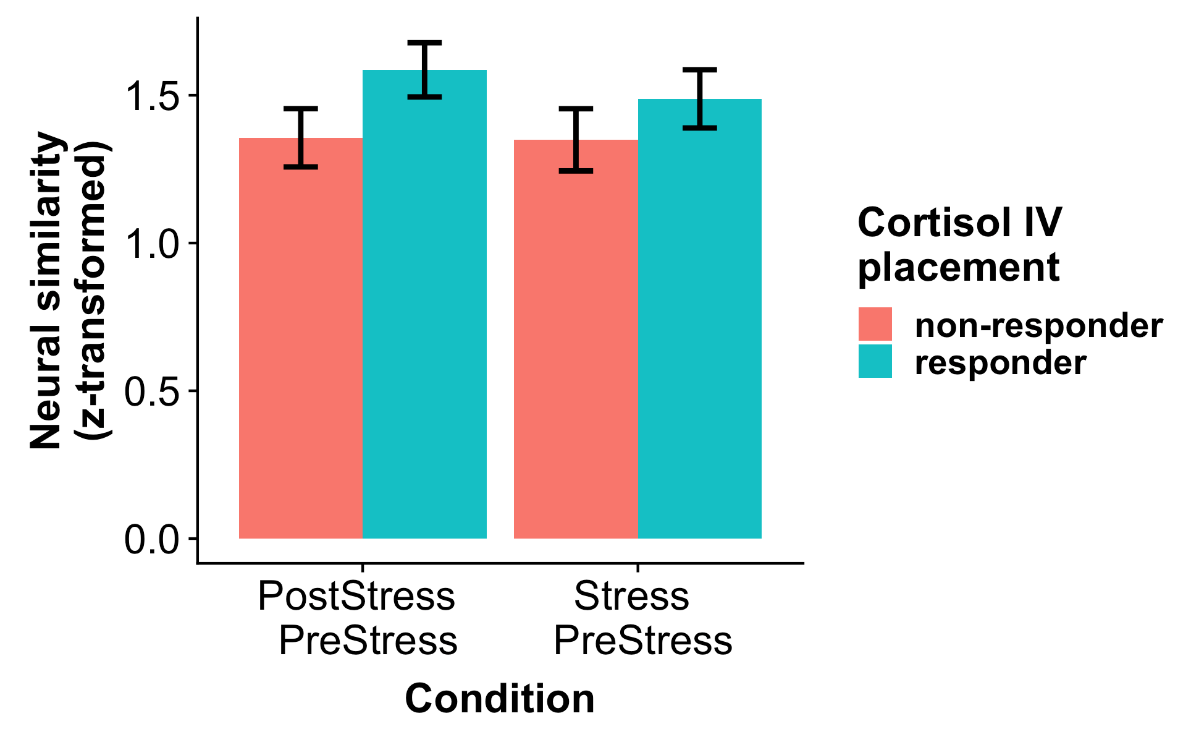


**Figure S2:** Intraindividual similarity between neural activity PreStress and PostStress is higher in IV placement responders compared to non-responders (b= 0.16, p= .024, CIboot= [0.02 - 0.30]). Y-axis depicts neural similarity (z-transformed correlation) between neural activity across regions of interest. Error bars indicate the 95% confidence intervals.

**
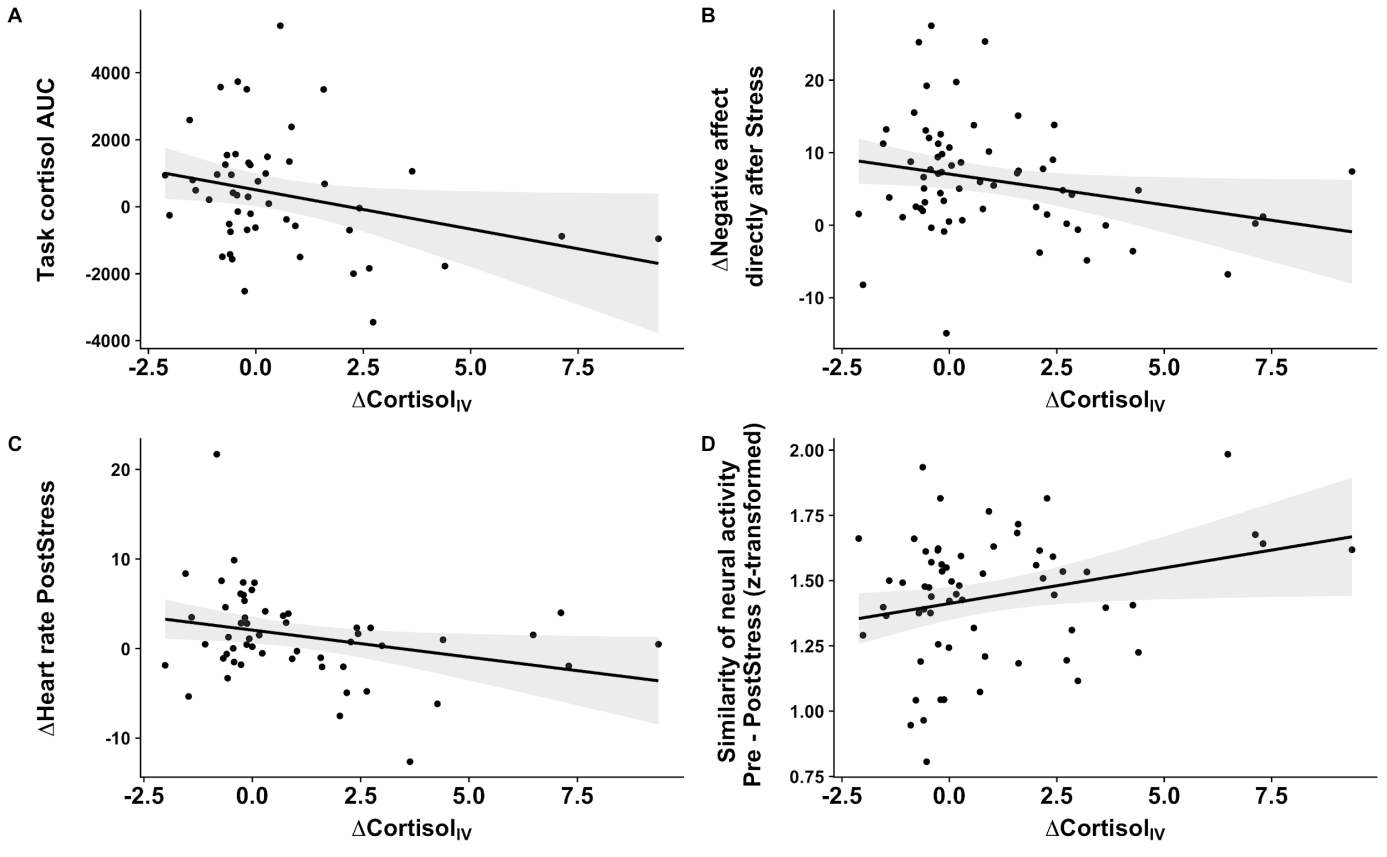
**

**Figure S3:** IV placement (IVP) induced cortisol (∆Cortisol_IVP_ always on the x-axis) increases influence the endocrine, autonomous, subjective, and neural response to the stress task. A) Cortisol response (Serum cortisol AUC) to the stress task is lower after high cortisol responses to IVP B) Negative affect (∆Negative at T6) is less increased after the stress task after high cortisol responses to IVP. C) Heart rate increases in the PostStress (∆HR_PostStress_) condition are reduced after high cortisol responses to IV placement. D) ROI similarity of neural activity Pre- and PostStress indicating stress recovery to baseline is higher after high cortisol responses to IVP. Linear regression lines are corrected for age, sex, and lifetime diagnosis status (and average FD for neural similarity). Shaded areas depict 95% confidence intervals.

## **Table**

**Table S1**: Current and lifetime prevalence of psychiatric disorders identified using the CIDI in the present sample.

|  | **12-months diagnosis**  **N(%)** | **Lifetime diagnosis**  **N(%)** |
| --- | --- | --- |
| Substance use disorders (F1) | 3 (4%) | 13 (19%) |
| Mood disorders (F3) | 4 (6%) | 5 (7%) |
| Anxiety-related disorders (F4) | 12 (18%) | 27 (40%) |
| Other disorders | 0 (0%) | 7 (6%) |
| No diagnoses | 51 (76%) | 32 (48%) |
| 1 diagnosis | 13 (19%) | 23.(34%) |
| 2 diagnoses | 2 (4%) | 9 (13%) |
| 3 and more diagnoses | 0 (0%) | 3 (4%) |

*Note:* Lifetime diagnosis status (dummy-coded yes/no) was used as a covariate in all analyses.
